# ADOB: A Field-Friendly Control Framework for Reliable Robotic Systems via Complementary Integration of Robust and Adaptive Control

**DOI:** 10.3390/s26051443

**Published:** 2026-02-25

**Authors:** Jangyeon Park, Kwanho Yu, Jungsu Choi

**Affiliations:** 1 Humanics Co., Ltd., Gyeongsan 38541, Republic of Korea; jypark@humanics.kr; 2 Department of Robotics Engineering, Yeungnam University, Gyeongsan 38541, Republic of Korea; kwanhoyu@yu.ac.kr

**Keywords:** adaptive control, complementary control, control framework, disturbance observer, model-based control, model uncertainty, motion tracking control, parameter adaptation: recursive least squares, robust control

## Abstract

Practical robotic systems require control methods that remain reliable under limited computational resources, uncertain environments, and frequent changes in operating conditions. Although model-based control forms the foundation of high-performance robotics, real-world deployment is often hindered by model uncertainty, time-varying dynamics, and costly identification. As a result, low-order and intuitive control schemes remain dominant, yet such approaches often fail to sustain consistent performance under disturbances and parameter variations. Robust and adaptive control provide representative paradigms to address this gap, where a Disturbance Observer (DOB) suppresses uncertainty through disturbance rejection and a Parameter Adaptation Algorithm (PAA) improves model fidelity through online identification. However, direct integration of a DOB and a PAA often introduces functional interference, including mutual masking between disturbance compensation and parameter estimation, which compromises closed-loop stability. This paper proposes an Adaptive Disturbance Observer (ADOB) that integrates a DOB with online parameter adaptation. The ADOB updates the nominal model of the DOB in real time using a Recursive Least Squares (RLS)-based PAA, while a dual-filtering structure separates disturbance rejection and parameter identification. Stability is analyzed using hyperstability theory, where a smoothing mechanism enforces the slowly varying parameter assumption. Experiments on a one-Degree-of-Freedom (DOF) electromagnetic actuator and a three-DOF robotic manipulator demonstrate reductions in model uncertainty and tracking error compared with a conventional DOB.

## 1. Introduction

As robotic technologies continue to proliferate in the industrial and service domains, advances in control theory have enabled increasingly stable and reliable operation [1,2,3,4,5,6]. Paradoxically, such theoretical progress has increased the complexity of control architectures, making implementation and tuning more challenging in practice and, in turn, widening the gap between theory and implementation [7,8]. Meanwhile, modern robotic control has expanded toward advanced methodologies, including model predictive control, continuous-time state estimation, and safe learning frameworks, further increasing design complexity and practical tuning requirements [3,5,6]. Consequently, in real-world environments where available resources are limited and environmental uncertainty is pervasive, simple and intuitive control schemes such as Proportional–Integral–Derivative (PID) control remain dominant over high-dimensional controllers [9,10]. Nevertheless, these approaches have inherent limitations in guaranteeing consistent performance under varying conditions, including changes in system dynamics, external disturbances, or changes in operators and operating conditions [9,10]. Therefore, for practical applicability in field environments, a controller should preserve ease of implementation and operation while achieving high reliability through mathematically grounded design.

A reliable control system should explicitly characterize causal relationships in the control process and provide quantitative assessments of performance and stability based on rigorous analysis. From this perspective, model-based control has been widely adopted as the core methodology for the precise control of robotic systems [1,7,8]. However, as structural complexity increases and operating environments become more demanding, the difficulty of model identification, which is a central step in model-based control, increases sharply and becomes a major barrier to practical implementation [11,12,13,14]. In particular, nonlinearities and time-varying characteristics inherent in the system degrade the predictability of the model, while persistent disturbances are superimposed on identification residuals and obscure physical causality [7,12,15]. Attempting to identify such a system directly, with the compound uncertainty left intact, is inefficient and often impractical [11,12,13]. From a pragmatic point of view, an effective alternative is to actively suppress uncertainty through inner-loop control to create an identification-friendly environment, while performing online model identification in parallel [12,16,17].

To enhance reliability under uncertainty in the system, two major approaches have been widely investigated: Robust control and Adaptive control. Robust control treats uncertainty as a disturbance to be suppressed, emphasizing stability guarantees under bounded uncertainty, often at the expense of conservative design [8,18]. In contrast, adaptive control interprets uncertainty as parameter variation to be accommodated through online model updates, while introducing additional estimation dynamics into the closed-loop system [19,20,21,22]. By integrating representative techniques of these paradigms, namely a Disturbance Observer (DOB) [16,17] and a Parameter Adaptation Algorithm (PAA), it becomes possible to manage nonlinearities, disturbances, and time-varying dynamics in a unified manner, thereby establishing an environment that is favorable for model identification. Specifically, the PAA updates the parameters of the DOB nominal model online based on real-time identification, yielding a Linear Time-Varying (LTV) model, while the DOB immediately compensates for disturbances and model mismatch using the updated model [16,17,19,21]. Within this structure, nonlinearities and disturbances are suppressed, while time-varying characteristics are tracked with high fidelity. As a result, the system maintains behavior consistent with a linear system, enabling continuous predictability and reliable control performance. In addition, an automated PAA identification process based on optimization algorithms such as Recursive Least Squares (RLS) or gradient-based methods removes the need for manual adjustment and improves implementability in practice [12,23,24].

An important point is that integration of robust control and adaptive control provides more than a simple functional addition; instead, it forms a strong positive feedback cycle in which inherent limitations of each method are alleviated by the other [25]. In conventional DOB design, a static nominal model is used; therefore, even time-varying characteristics of the system are effectively treated as disturbances, and the resulting unavoidable model mismatch imposes a constraint that forces a conservative design of the bandwidth of the Q-filter to ensure stability [16,17]. In addition, when a PAA is exposed to disturbances and uncertainty, an adaptation gain is often kept low to prevent oscillation or divergence of parameter estimates, which sacrifices responsiveness to parameter variation [19,20,21]. Under these conditions, hybrid integration becomes a key mechanism that releases mutual design constraints. When the PAA updates the nominal model of the DOB according to real-time parameter estimation and aligns the nominal model with the dynamics of the system, the DOB is relieved from compensating time-varying characteristics as disturbances and gains sufficient margin to substantially expand the Q-filter bandwidth [16,17]. At the same time, when the DOB suppresses nonlinearities and disturbances in a proactive manner and stabilizes the behavior of the system within the validity range of the nominal model, the PAA is provided with refined data that enable a more aggressive adaptation gain, thus improving both the convergence speed and the accuracy of the estimation [19,20,21]. Consequently, this integrated strategy establishes a synergistic interaction in which robust control creates a stable environment for parameter adaptation, while adaptive control continuously updates the nominal model that underpins robust control. This positive feedback structure extends the attainable performance of model-based control and enables high control quality in which precision and robustness coexist, even under complex operating environments.

In this paper, an Adaptive Disturbance Observer (ADOB) is proposed to maximize the control performance of robotic systems by integrating a nominal-model update mechanism based on real-time parameter estimation. The ADOB realizes the positive feedback cycle described above by projecting time-varying characteristics identified by the PAA onto the nominal model of the DOB. However, when the two mechanisms are combined in a naive parallel manner, functional interference may occur, such as misinterpretation of compensation signals as disturbances or overlap of operational bandwidths that compromises stability of the control loop [16,17,19,21]. To address this issue, this study introduces a dual-filtering mechanism that systematically separates and complements the roles of each algorithm, thus ensuring operational stability of the hybrid structure. With this enhancement, a field engineer can deploy the ADOB by applying a coarse initial model established from simple excitation inputs. During operation, the DOB immediately compensates for model mismatch to ensure stability, while the PAA gradually refines the nominal model toward an optimal LTV model that conforms to the dynamics of the system. Consequently, a user can apply high-performance linear model-based control algorithms, such as Zero Phase Error Tracking Control (ZPETC) [26], Linear Quadratic Regulator (LQR) [27], or Model Predictive Control (MPC) [28], within a predictable linear environment maintained by the ADOB, without the additional burden of complex identification procedures or iterative trial-and-error tuning.

## 2. An Adaptive Disturbance Observer

### 2.1. Structure of ADOB

**Figure 1:**
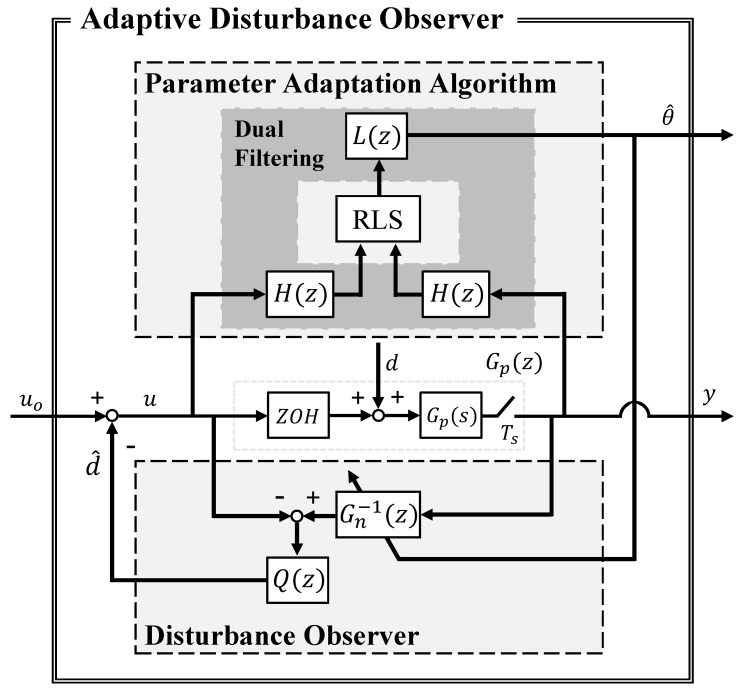
Structure of the ADOB.

The proposed ADOB combines robust control based on the DOB with the PAA to maximize synergy between disturbance rejection and online model adaptation within an integrated framework. Figure 1 illustrates the overall block diagram of the ADOB, where the definitions of variables and blocks are summarized as follows: $u_{o},u\in R^{m}$ denote an external control input and an input of the system, respectively, and $y\in R^{p}$ denotes an output of the system. Correspondingly, $d,\hat{d}\in R^{m}$ denote an actual disturbance and an estimated disturbance, respectively. To facilitate the decentralized control of complex robotic systems and ensure practical implementability, this study adopts localized Single-Input Single-Output (SISO) configuration where m=p=1 are utilized for each independent joint controller. The plant dynamics are represented by $G_{p}\left(s\right)$ in the continuous-time domain and by $G_{p}\left(z\right)$ in the discrete-time domain with sampling time $T_{s}$ and Zero-Order Hold (ZOH). The variable $G_{n}\left(z\right)$ denotes the nominal model, and Q(z) denotes the Q-filter that is a core element of the DOB.

In the block diagram, the DOB in the lower path estimates and compensates for disturbances and model mismatch based on the nominal model and input–output data of the system. Meanwhile, the PAA on the upper path performs real-time identification using the RLS algorithm and immediately reflects the identification result in the nominal model $G_{n}\left(z\right)$ of the DOB to maintain the validity of the nominal model [12,23]. In this process, the dual-filtering is applied to the input and output paths of the RLS so that functional interference between compensation of the DOB and identification of the PAA is systematically separated and stability of the overall loop is ensured.

### 2.2. Nominal Modeling of the System via DOB

To analyze the estimated disturbance captured by the DOB, the relationship between the actual plant and its inherent uncertainties can be defined as follows:

(1) $$G_{p}\left(z\right)=G_{n}\left(z\right)\left(1+\Delta \left(z\right)\right)$$

In this context, Δ(z) is denoted as the uncertainty of the plant, applied in a multiplicative manner to reflect realistic environments. It is worth noting that the initial nominal model must be stable. This assumption deserves clarification because many physical setups exhibit open-loop instability, and representing every system as in (1) might be considered unsuitable. However, this issue can be addressed by applying a stabilizing controller to form a closed-loop system; the resulting closed-loop transfer function can then be interpreted as the nominal model. Assuming the initial plant is in a stable state as shown in Figure 1, the disturbance $\hat{d}$ estimated through the DOB inner loop is defined as follows:

(2) $$\hat{d}\left(k\right)=Q\left(z\right)\left[\left(G_{n}^{-1}\left(z\right)-G_{p}^{-1}\left(z\right)\right)y\left(k\right)+d\left(k\right)\right]$$

Here, Q(z) is a filter used for the order compensation required for implementation of $G_{n}^{-1}\left(z\right)$ and to mitigate the instability induced by the mismatch of the model, and is typically implemented as a Low-Pass Filter (LPF). More specifically, according to the Small Gain Theorem, Q(z) should be designed to satisfy the following condition for stable operation of the DOB:

(3) $$∥Q\left(e^{j\omega T}\right)\Delta \left(e^{j\omega T}\right){∥}_{\infty }<1$$

When robust stability of the system is ensured through the designed Q(z), the DOB can immediately compensate the estimated disturbance $\hat{d}$ and feed the compensation into the control loop, as illustrated in Figure 1. The compensation signal $\hat{d}$ includes not only external disturbances, but also mismatch components between the actual system and the nominal model. Therefore, through this cancellation process, the DOB effectively drives the actual plant to follow the designed nominal model, thus maintaining predictability throughout the operation [16,17].

### 2.3. Nominal Model Update of DOB via PAA

Time-varying characteristics caused by heat generation or component wear induce inevitable model mismatch in conventional DOB structures that rely on a static nominal model. This structural limitation leads to conservative Q-filter bandwidth design to ensure stability, which degrades performance of disturbance estimation as a consequence. This limitation can be alleviated by updating the nominal model in real time via the PAA. When dynamics of the system are expressed as a vector-form difference equation, the following relation is obtained:

(4) $$u\left(k\right)=\theta^{T}\phi \left(k+r\right)$$

where $\theta \in R^{n}$ and $\phi \left(k\right)\in R^{n}$ denote the parameter vector and the regressor vector, respectively, where *n* is the number of identified model parameters, and *r* denotes the relative degree of the transfer function $G_{n}\left(z\right)$. The DOB estimates disturbances by comparing an input of control with an estimated input derived from the inverse nominal model. If model parameters were identified accurately and issues of physical realizability were ignored, the disturbance could be computed as $d\left(k\right)=\theta^{T}\phi \left(k+r\right)-u\left(k\right)$ as implied by (2). However, in practice, these parameters are uncertain and often exhibit time-varying behavior. Therefore, a more reliable disturbance estimate is expressed as:

(5) $$\hat{d}\left(k\right)={\hat{\theta }}^{T}\phi \left(k+r\right)-u\left(k\right)$$

where $\hat{\theta }$ denotes an optimal estimate of θ obtained via least-squares using input–output data.

RLS is employed to minimize the $\ell_{2}$-norm of the estimated disturbance while incorporating a forgetting factor:

(6) $$J\left(k\right)=\sum_{i=r}^{k}\lambda^{k-i}{\hat{d}}^{T}\left(i-r\right)\hat{d}\left(i-r\right)$$

where λ (0≤λ≤1) represents the forgetting factor, and $\hat{d}$ is defined as in (5) [23,29]. The term $\hat{d}\left(i-r\right)$ is utilized in the cost function to account for the dependency of $\hat{d}\left(i\right)$ on future output data y(i+r). The stability of this recursive adaptation process is established by considering the PAA as part of an Equivalent Feedback System (EFS). As will be detailed in Section 3.1, the convergence of the disturbance estimate $\hat{d}\left(k\right)$ is rigorously guaranteed by satisfying the hyperstability conditions, specifically the Strictly Positive Real (SPR) property and the Popov integral inequality.

Inserting (5) into (6) results in the following expression:

(7) $$J\left(k\right)=\sum_{i=r}^{k}\lambda^{k-i}{\left(u\left(i-r\right)-{\hat{\theta }}^{T}\left(k\right)\phi \left(i\right)\right)}^{2}$$

This performance index mirrors standard indices used in parameter adaptation, with the specific distinction of using the input u(i−r) instead of the output y(i). Consequently, the adaptation law is derived according to the generalized RLS theorem:

(8) $$\hat{\theta }\left(k\right)=\hat{\theta }\left(k-1\right)+F\left(k\right)\phi \left(k\right)\left[u\left(k-r\right)-{\hat{\theta }}^{T}\left(k-1\right)\phi \left(k\right)\right]$$

where the gain matrix $F\left(k\right)\in R^{n\times n}$ is updated as:

(9) $$F\left(k\right)=\frac{1}{\lambda }\left[F\left(k-1\right)-\frac{F\left(k-1\right)\phi \left(k\right)\phi^{T}\left(k\right)F\left(k-1\right)}{\lambda +\phi^{T}\left(k\right)F\left(k-1\right)\phi \left(k\right)}\right]$$

with an arbitrary positive definite initial matrix F(0).

In particular, by applying the Sherman–Morrison formula [23,29,30], also known as the Matrix Inversion Lemma, the complex update law can be simplified into the compact recursive form shown in (8) and (9). This formulation allows the PAA to avoid direct matrix inversion, effectively reducing the computational complexity to $O(n^{2})$ per update. Combined with the localized SISO configuration (m=p=1) introduced in Section 2.1, this efficient recursive structure minimizes the computational overhead and ensures stable real-time implementation even in relatively resource-constrained embedded control environments.

While the RLS-based PAA progressively reduces the estimated disturbance $\hat{d}$ through online adjustment of the nominal model, it is important to clarify that $\hat{d}\to 0$ does not necessarily imply $\tilde{\theta }\to 0$, i.e., exact convergence of the parameter estimation error is neither required nor pursued in the proposed framework. The objective of ADOB is not rigorous system identification in the classical sense, but the continual adaptation of model parameters toward values that are consistent with the control objective. In other words, the adaptation seeks a control-relevant parameterization rather than the true physical parameters of the plant.

From this perspective, effective operation of the RLS mechanism in ADOB can be achieved without requiring strict satisfaction of the Persistence-of-Excitation (PE) condition. Even in the absence of sufficient excitation for parameter convergence, the reduction in $\hat{d}$ indicates that the updated nominal model is adequately capturing the dynamics that are relevant to disturbance estimation and compensation. The adaptation law therefore continues to adjust the parameters so as to minimize the modeling error seen in the DOB, ensuring that the controller operates with an effectively matched model at each instant. This interpretation aligns with a hyperstability-based viewpoint, where the adaptation remains bounded and functionally meaningful without requiring asymptotic identification. Nevertheless, under insufficient excitation, the RLS estimator does not simply halt adaptation; rather, the regression becomes ill-conditioned, leading to numerically amplified gain updates and consequently to oscillatory or fluctuating parameter estimates.

### 2.4. Functional Harmonization of DOB and PAA via Dual-Filtering

The ADOB framework integrates robust control and adaptive control; however, both approaches use information about model mismatch while pursuing fundamentally different objectives. The DOB interprets this information as a disturbance to be rejected, whereas the PAA exploits the same information as an informative signal for tracking variations in the parameters of the system. This mismatch of objectives inevitably induces functional conflict within a single framework [16,17,19,20]. More specifically, as the DOB compensates errors more effectively, the RLS prediction error term in (8), namely $u\left(k-r\right)-{\hat{\theta }}^{T}\left(k-1\right)\phi \left(k\right)$, tends to vanish, which yields a masking effect. As a consequence, the PAA cannot acquire informative data for learning even when the true parameters change, and the identification function fails to operate as intended. Therefore, to prevent interference between the two functions and to create complementary synergy, the ADOB applies a dual-filtering scheme to the input and output paths of the RLS and functionally separates the two algorithms.

#### 2.4.1. H(z): Washout Filter for Frequency-Domain Decoupling

The washout filter H(z) is placed in the RLS input path to ensure reliable parameter identification even in the presence of disturbances. As illustrated in Figure 1, the output *y* is affected by both the control input *u* and the disturbance *d*, which yields $Y\left(z\right)=G_{p}\left(z\right)\left[U\left(z\right)+D\left(z\right)\right]$. To suppress the disturbance component and prevent degradation of identification performance, the ADOB applies H(z) to both sides of the relation and obtains the following filtered relationship:

(10) $$H\left(z\right)Y\left(z\right)=G_{p}\left(z\right)H\left(z\right)U\left(z\right)+G_{p}\left(z\right)H\left(z\right)D\left(z\right)$$

The filter H(z) should be designed so that the noise-like components of the disturbance are effectively attenuated and the following condition is satisfied:

(11) $$\left|G_{p}\left(e^{j\omega T}\right)H\left(e^{j\omega T}\right)D\left(e^{j\omega T}\right)\right|\approx 0$$

Given that Q(z) in the DOB is typically implemented as an LPF, design of H(z) as a High-Pass Filter (HPF) induces functional decoupling between the DOB and the PAA in the frequency domain. This strategic allocation ensures that each algorithm operates in an appropriate frequency band without mutual interference; as a result, the PAA can identify the true parameter vector θ even in environments with significant disturbances.

#### 2.4.2. L(z): Smoothing Filter for Stabilizing Parameter Convergence

The stability of the ADOB inherits the robust stability condition of a conventional DOB under the assumption that parameter variations occur sufficiently slowly. According to the Small Gain Theorem, stability is guaranteed when the model uncertainty Δ(z) in (1) satisfies the condition in (3). The proposed structure actively expands the stability margin by minimizing Δ(z) through real-time updates of the nominal model $G_{n}\left(z,\hat{\theta }\right)$ via the PAA.

However, abrupt variations in the parameter estimates $\hat{\theta }$ identified by the RLS algorithm intensify the time-varying characteristics of the system, thus undermining the time-invariant analysis framework assumed by the Small Gain Theorem. Additionally, when the PE condition is insufficiently satisfied, the regression becomes poorly conditioned, and the RLS update may produce fluctuation or oscillatory behavior in the estimated parameters rather than meaningful convergence. Such time-varying behavior obscures the definition of frequency responses and may yield divergent transient responses. To mitigate this issue, an LPF, L(z), is placed in the output of the RLS algorithm to physically constrain the rate of parameter updates.

The filter L(z) suppresses high-frequency fluctuations in the parameter estimates and promotes smooth convergence, while enforcing time-scale separation between the DOB and the PAA. Specifically, by allowing only gradual parameter variations, the DOB is provided with sufficient temporal margin to promptly compensate for short-term disturbances. As a result, the estimated parameters are incorporated into the nominal model only after stable convergence, thus ensuring both robustness and dynamic accuracy throughout the adaptation process.

#### 2.4.3. Design Guidelines for ADOB Filters

To facilitate the successful field implementation of the ADOB, this section suggests a practical design procedure for the filters Q(z), H(z), and L(z). Focusing on their functional roles, the design criteria are as follows:

1. Q(z): Rejection of External Disturbances and Model Discrepancy
  - Initial Setup: Establish a rough nominal model through simple experiments, as high initial precision is not required. Based on this, set the LPF cutoff frequency as high as possible while satisfying $∥Q\left(z\right)\Delta \left(z\right){∥}_{\infty }<1$ to maximize initial performance.
  - Tuning Strategy: While bandwidth can be reduced for stability, the PAA reduces model discrepancy and expands the stability margin. Thus, tuning toward a higher bandwidth is recommended to maximize disturbance rejection and tracking precision.
2. H(z): Frequency-Domain Decoupling between Disturbance Rejection and Adaptation
  - Initial Setup: Identify the frequency range of dominant disturbances such as gravity and friction. As these are typically concentrated in the low-frequency region, a cutoff frequency of 5 Hz, for instance, can serve as a representative initial boundary. Setting the HPF cutoff at this point physically masks disturbances from the PAA to prevent estimation bias.
  - Tuning Strategy: While the cutoff frequency balances resolution and masking, raising it is recommended to minimize interference between the DOB and PAA. This ensures clear functional separation by limiting PAA operation to a frequency range beyond the primary disturbance rejection band of the DOB.
3. L(z): Smoothing of Parameter Adaptation and Time-Scale Separation
  - Initial Setup: Set the LPF cutoff frequency at approximately 10–20% of the closed-loop control bandwidth. This relatively high initial bandwidth ensures rapid convergence by quickly absorbing initial modeling errors.
  - Tuning Strategy: If the parameter update is perceived as sluggish, the cutoff frequency may be increased to accelerate adaptation. However, maintaining a lower bandwidth is generally recommended for long-term operational stability and effective noise filtering, reinforcing the slowly varying parameter assumption.

## 3. Stability and Performance Analysis of the ADOB

In this section, the control stability of the proposed ADOB is theoretically verified, and its superior performance is analyzed through comparison with the standalone DOB and PAA implementations.

### 3.1. Control Stability

The stability evaluation of the ADOB is performed using hyperstability theory [31,32], which is widely used to establish the convergence of the PAA. First, the *a priori* and *a posteriori* disturbance estimates that form the basis of the stability analysis are defined as follows:

(12) $$\begin{array}{c} a\;\;priori:{\hat{d}}^{o}\left(k-r\right)={\hat{\theta }}^{T}\left(k-1\right)\phi \left(k\right)-u\left(k-r\right)\\ a\;\;posteriori:\hat{d}\left(k-r\right)={\hat{\theta }}^{T}\left(k\right)\phi \left(k\right)-u\left(k-r\right) \end{array}$$

By applying the definition in (12) together with the recursive formulation established in Section 2.3 using the Matrix Inversion Lemma, the complex update law in (8) can be simplified into a compact recursive form based on the *a posteriori* estimate:

(13) $$\hat{\theta }\left(k\right)=\hat{\theta }\left(k-1\right)-F\left(k-1\right)\phi \left(k\right)\hat{d}\left(k-r\right)$$

For stability analysis, when the true parameter vector $\theta \in R^{n}$ is assumed to be constant or to vary sufficiently slowly, the dynamics of the parameter estimation error vector $\tilde{\theta }\left(k\right)\in R^{n}$, defined as $\tilde{\theta }=\hat{\theta }-\theta$, are governed by:

(14) $$\tilde{\theta }\left(k\right)=\tilde{\theta }\left(k-1\right)-F\left(k-1\right)\phi \left(k\right)\hat{d}\left(k-r\right)$$

Considering that $u\left(k-r\right)=\theta^{T}\phi \left(k\right)$, the *a posteriori* disturbance estimate in (12) can be reformulated in terms of the parameter estimation error as:

(15) $$\hat{d}\left(k-r\right)={\tilde{\theta }}^{T}\left(k\right)\phi \left(k\right)$$

As shown in (14) and (15), $\tilde{\theta }\left(k\right)$ and $\hat{d}\left(k-r\right)$ interact within a closed-loop feedback structure, forming an EFS composed of a linear block and a nonlinear block. To analyze the asymptotic stability of this error system, the external input is assumed to be zero. According to hyperstability theory, stability is guaranteed if the linear block is SPR and the nonlinear block satisfies the Popov integral inequality [31].

In this framework, the forward linear block processes the input signal ${\tilde{\theta }}^{T}\left(k\right)\phi \left(k\right)$ to generate the a posteriori disturbance estimate $\hat{d}\left(k-r\right)$ as its output. Since the ADOB is designed such that $\hat{d}\left(k-r\right)={\tilde{\theta }}^{T}\left(k\right)\phi \left(k\right)$ as shown in (15), the transfer function of the linear block is simply the identity $H_{L}\left(z\right)=I$. Such an identity transfer function inherently satisfies the SPR condition because its phase lag remains at zero for all frequencies.

Simultaneously, the nonlinear block, representing the RLS-based adaptation law in (14), takes the estimation error $\hat{d}\left(k-r\right)$ as its input to produce the parameter error feedback $-{\tilde{\theta }}^{T}\left(k\right)\phi \left(k\right)$. By defining a Lyapunov-like energy function $V\left(k\right)={\tilde{\theta }}^{T}\left(k\right)F^{-1}\left(k\right)\tilde{\theta }\left(k\right)$, the recursive minimization of the cost function ensures that the energy remains bounded. This leads to the satisfaction of the Popov summation for any arbitrary finite discrete-time $k_{1}\geq 0$:

(16) $$\sum_{k=0}^{k_{1}}\hat{d}\left(k-r\right)\left[-{\tilde{\theta }}^{T}\left(k\right)\phi \left(k\right)\right]=-\sum_{k=0}^{k_{1}}{\hat{d}}^{2}\left(k-r\right)\geq -V\left(0\right)$$

where $V\left(0\right)=\delta_{0}^{2}$ is a finite positive constant determined by the initial parameter error and adaptation gain.

By fulfilling both the SPR and Popov conditions, the disturbance estimate $\hat{d}\left(k\right)$ is guaranteed to converge to zero, ensuring the effective minimization of model uncertainty. While exact parameter convergence ($\tilde{\theta }\to 0$) remains a significant metric in general system identification, the proposed ADOB is specifically designed to prioritize the elimination of the modeling-induced disturbance $\hat{d}\left(k\right)$ for control-relevant adaptation. This focus allows the nominal model to remain sufficiently matched for compensation even during phases where parameter convergence is incomplete. Furthermore, the smoothing filter L(z) reinforces the slowly varying parameter assumption by mitigating numerical fluctuations during periods of limited excitation, thus sustaining the validity of the hyperstability framework in practical field environments.

### 3.2. Control Performance

To evaluate the control performance, let *r* denote the residual within the closed-loop architecture and *e* represent the performance error induced by this residual. In this section, ${∥\cdot ∥}_{2}$ denotes the $\ell_{2}$ norm of a discrete-time signal, i.e., ${∥x∥}_{2}:={\left(\sum_{k=0}^{\infty }x{\left(k\right)}^{2}\right)}^{1/2}$.

Assuming that the closed-loop transfer from *r* to *e* is internally stable, the induced $\ell_{2}$ gain of this transfer is finite. Let

(17) $$\kappa :=∥T_{e\leftarrow r}{∥}_{\infty }\in R_{>0}$$

denote the induced $H_{\infty }$ norm of the closed-loop mapping from *r* to *e*, which signifies the induced $\ell_{2}$ gain of the system. By the definition of induced norm, the following inequality holds:

(18) $${∥e∥}_{2}\leq \kappa {∥r∥}_{2}$$

The residual is defined as r:=d+ΔGu, where $\Delta G:=G_{p}-G_{n}$ represents the discrepancy between the physical plant dynamics and the nominal model. Applying the triangle inequality yields ${∥r∥}_{2}\leq {∥d∥}_{2}+∥\Delta Gu{∥}_{2}$.

To quantify the effectiveness of each control component, we define $\gamma_{\mathrm{DOB}}$ and $\gamma_{\mathrm{PAA}}$ as the attenuation coefficients for the DOB and PAA, respectively. These coefficients are scalar values representing the suppression capability of each mechanism and satisfy $0<\gamma_{\mathrm{DOB}},\gamma_{\mathrm{PAA}}<1$. More precisely, $\gamma_{\mathrm{DOB}}$ and $\gamma_{\mathrm{PAA}}$ represent norm-reduction factors applied to the disturbance component and the model-mismatch component of the residual, respectively.

In a single-loop DOB configuration, the disturbance-related component of the residual is attenuated by a factor of $\left(1-\gamma_{\mathrm{DOB}}\right)$. Substituting this attenuation into (18) gives the performance bound

(19) $${∥e_{\mathrm{DOB}}∥}_{2}\leq \kappa \left(1-\gamma_{\mathrm{DOB}}\right)\left({∥d∥}_{2}+{∥\Delta Gu∥}_{2}\right)$$

In contrast, stand-alone PAA mitigates only modeling errors, yielding

(20) $${∥e_{\mathrm{PAA}}∥}_{2}\leq \kappa \left({∥d∥}_{2}+\left(1-\gamma_{\mathrm{PAA}}\right){∥\Delta Gu∥}_{2}\right)$$

In the proposed ADOB framework, parameter adaptation and disturbance observation operate sequentially on the same residual. Parameter adaptation first reduces the modeling-error component, and the DOB subsequently suppresses the remaining residual. Applying the attenuation factors sequentially in the induced-norm bound (18) yields

(21) $${∥e_{\mathrm{ADOB}}∥}_{2}\leq \kappa \left(\left(1-\gamma_{\mathrm{DOB}}\right){∥d∥}_{2}+\left(1-\gamma_{\mathrm{DOB}}\right)\left(1-\gamma_{\mathrm{PAA}}\right){∥\Delta Gu∥}_{2}\right)$$

Accordingly, under the same induced-gain κ, the ADOB performance satisfies

(22) $${∥e_{\mathrm{ADOB}}∥}_{2}\leq \min\left\{\;∥e_{\mathrm{DOB}}{∥}_{2},\;{∥e_{\mathrm{PAA}}∥}_{2}\;\right\}$$

The above inequalities follow directly from the induced-norm properties of stable linear operators and standard norm inequalities, thereby providing a deterministic performance bound for each configuration.

## 4. Experimental Results with a One-DOF Electromagnetic Actuator

The experimental setup was configured in a simplified form to reduce structural side effects and to clearly observe the inherent behavior of the control algorithms. The system was restricted to a one-Degree-of-Freedom (DOF) to enable a focused investigation of the effects of model uncertainty and parameter variations on control performance. In this configuration, both the conventional DOB and the proposed ADOB were evaluated under strictly identical conditions, supporting a more objective evaluation of the strengths and limitations of the proposed ADOB.

### 4.1. Experimental Setup

**Figure 2:**
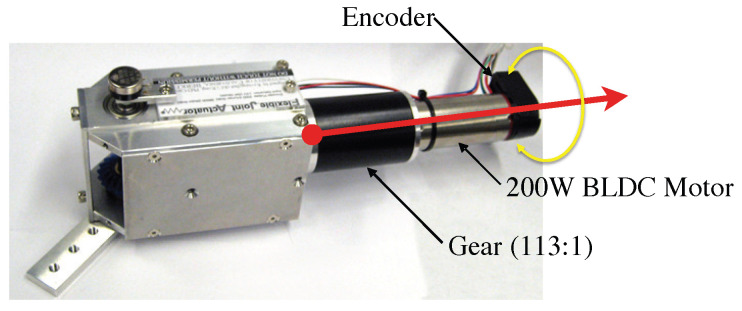
Experimental testbed 1; BLDC motor with gear train.

To validate the efficacy of the proposed control strategy, an experimental testbed featuring a one-DOF electromagnetic actuator for a robotic manipulator was established. As illustrated in Figure 2, the hardware setup comprises a 200 W brushless DC motor (Maxon EC30 PowerMax, Sachseln, Switzerland) (Maxon EC30 PowerMax) integrated with a 113:1 high-reduction gearbox. The closed-loop control system operates at a sampling frequency of 1 kHz, with inherent modeling uncertainties such as friction and backlash included to challenge the controller’s robustness. In particular, to explicitly assess the parameter adaptation capabilities of the ADOB, a conservative operating environment was simulated by manually adjusting the servo amplifier dial to induce rapid fluctuations in the motor torque gain, $K_{T}$, within a brief operational window.

**Figure 3:**
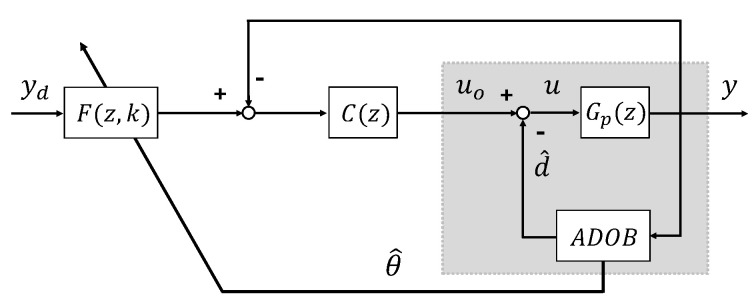
Block diagram of multi degrees-of-freedom control with the ADOB.

To achieve high-precision tracking control, a multi-DOF control architecture integrating a feedback controller and a feedforward filter was designed as shown in Figure 3. A Proportional–Derivative (PD) controller was utilized for the feedback component C(z) to facilitate straightforward implementation. The feedforward filter F(z,k), which governs trajectory-tracking accuracy, is based on the Perfect Tracking (PT) technique [26] and is defined as the inverse of the closed-loop transfer function as follows:

(23) $$F\left(z,k\right)={\left[\frac{C\left(z\right)G_{n}\left(z,k\right)}{1+C\left(z\right)G_{n}\left(z,k\right)}\right]}^{-1}$$

**Figure 4:**
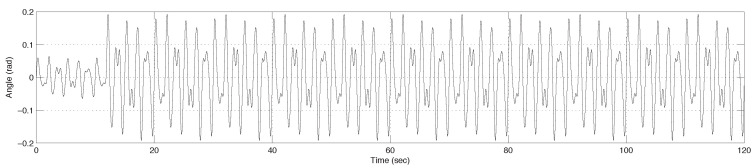
Designed reference angle trajectory for the one-DOF electromagnetic actuator system.

A pivotal characteristic of F(z,k) is the capability to update the nominal model $G_{n}\left(z,k\right)$ instantaneously using the real-time parameter estimates $\hat{\theta }$ provided by the ADOB. This mechanism allows a physical alteration in the system to be immediately reflected in the control model. The experimental objective is to demonstrate the capability of the system to precisely follow the complex sinusoidal reference trajectory in Figure 4, comprising components at 1.2, 2.0, and 3.0 Hz, despite significant parameter variation and nonlinear disturbances.

### 4.2. Controller Design

To evaluate the efficacy of the ADOB, tracking performance was benchmarked against a conventional DOB system. The Q-filter was designed to satisfy stability conditions through preliminary testing without manual servo amplifier adjustments, resulting in the following discrete-time transfer function:

(24) $$Q\left(z\right)=\frac{0.03634z+0.03634}{z^{2}-1.461z+0.5335}$$

**Figure 5:**
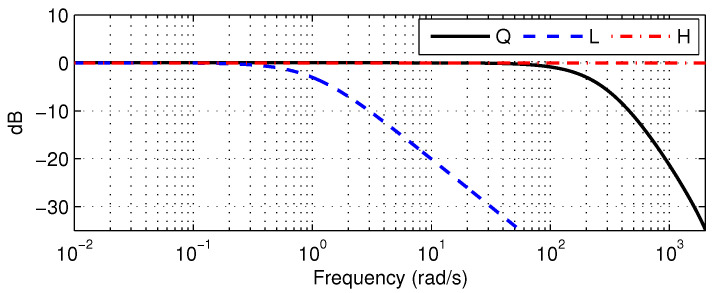
Frequency responses of the Q(z), L(z), and H(z).

As depicted in Figure 5, this design acts as an LPF with a 30 Hz cutoff frequency, which is wider than the bandwidth required for the designed reference trajectory. In the initial phase, the nominal model was established as:

(25) $$G_{n}\left(z\right)=\frac{1}{110,000z^{2}-220,000z+110,000}$$

This model served as the baseline for the update mechanism of the PAA, with the initial parameters set to $b_{0}$ = 11,000, $b_{1}$ = −22,000, and $b_{2}$ = 110,000. After establishment of the initial nominal model, a discrete-time PD controller was synthesized based on the nominal plant using the backward difference approximation. The controller was tuned to ensure robust stability by maintaining adequate gain and phase margins, which yields the following discrete-time expression:

(26) $$C\left(z\right)=\frac{1020z-979.6}{z}$$

To finalize the ADOB architecture, the PAA integrated with L(z) is designed to mitigate the dynamical impact of parameter updates, defined as follows:

(27) $$L\left(z\right)=\frac{0.001}{z-0.999}$$

### 4.3. PAA in the ADOB

**Figure 6:**
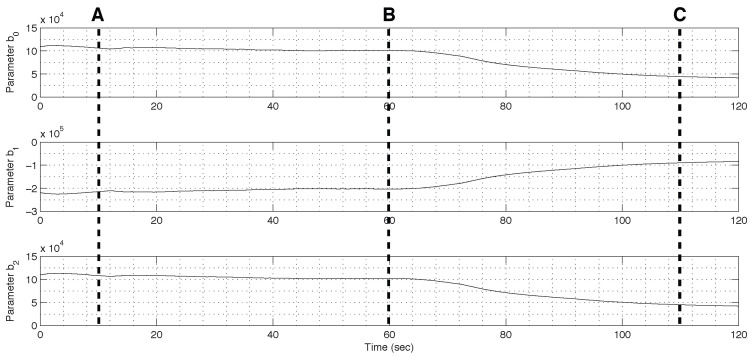
Plots for nominal model parameters ($G_{n}=\frac{1}{b_{2}z^{2}+b_{1}z+b_{0}}$) updated by the PAA over 120 s.

As illustrated in Figure 5, this filter has a cutoff frequency of 0.159 Hz. Within this experimental framework, the refined environment and streamlined configuration of the system are assumed to be the primary sources of disturbance; therefore, the washout filter H(z) was omitted in this study. This setup facilitates the assessment of the ADOB capability to handle model uncertainty in LTV systems relative to the conventional DOB. After completion of the overall controller configuration for motion tracking of the electromagnetic actuator, an artificial increase in the motor torque gain $K_{T}$ was induced after time B. Figure 6 depicts the resulting time-varying model parameters estimated by the PAA based on the nominal model structure.

**Figure 7:**
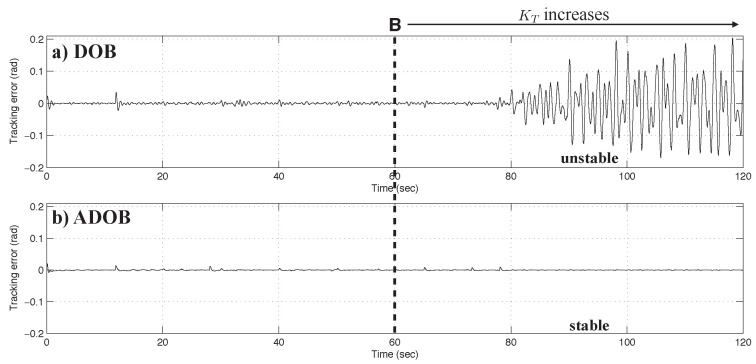
Measured tracking error (rad): (**a**) with the conventional DOB and (**b**) with the proposed ADOB.

The performance comparison of the DOB with the initial model and the ADOB with the updated model is illustrated in Figure 7.

### 4.4. Model Uncertainty with ADOB

Recall that the sufficient condition for the DOB to stably reject disturbances and model uncertainties is given by (3), which stipulates that the magnitude of Q(z) remains below the inverse of the model uncertainty (i.e., $|\Delta {\left(e^{j\omega T}\right)}^{-1}|$) across the entire frequency range. In this experiment, the magnitude of the inverse model uncertainty is observed to evaluate the performance of the proposed ADOB.

**Figure 8:**
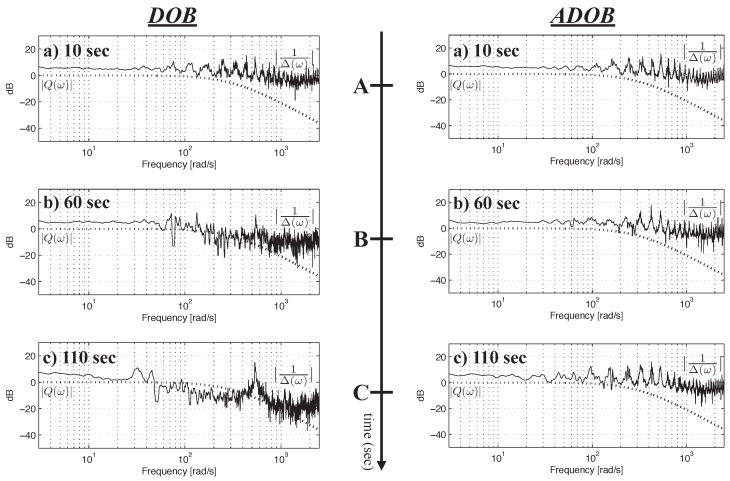
Frequency-response magnitude plots of $\left|Q(e^{j\omega T})\right|$ and $|\frac{1}{\Delta (e^{j\omega T})}|$ for the conventional DOB (**left**) and the proposed ADOB (**right**), evaluated at time instants A, B, and C corresponding to Figure 6.

Rearranging (1), the model uncertainty for a given $G_{p}\left(z\right)$ and $G_{n}\left(z\right)$ is defined as

(28) $$\Delta \left(\omega ,G_{p}\left(e^{j\omega T}\right),G_{n}\left(e^{j\omega T}\right)\right)=\frac{G_{p}\left(e^{j\omega T}\right)-G_{n}\left(e^{j\omega T}\right)}{G_{n}\left(e^{j\omega T}\right)}$$

where $G_{p}\left(e^{j\omega T}\right)$ and $G_{n}\left(e^{j\omega T}\right)$ represent the frequency-domain representations of $G_{p}\left(z\right)$ and $G_{n}\left(z\right)$, respectively. The nominal frequency response $G_{n}\left(e^{j\omega T}\right)$ is obtained analytically from the nominal model parameters, whereas $G_{p}\left(e^{j\omega T}\right)$ is computed numerically via Fourier transforms of the measured input and output signals. In this experiment, the **fft.m** function in MATLAB R2024a was applied to 2000 signal samples around the time instants of interest. Figure 8 illustrates the experimental results from the perspective of the Q-filter stability condition. The left column corresponds to the conventional DOB employing a fixed nominal model, while the right column represents the proposed ADOB incorporating online parameter adaptation. In both columns, the magnitudes of $\left|Q(e^{j\omega T})\right|$ and $|\Delta {\left(e^{j\omega T}\right)}^{-1}|$ are shown for direct comparison. The markers A, B, and C along the time axis indicate the same time instants as A, B, and C in Figure 6, respectively. For the conventional DOB, it is observed that $|\Delta {\left(e^{j\omega T}\right)}^{-1}|$ falls below $\left|Q(e^{j\omega T})\right|$ at t=60 s, violating the necessary stability condition of the DOB loop. Since a small $|\Delta {\left(e^{j\omega T}\right)}^{-1}|$ indicates substantial model uncertainty, this result implies that the dynamics of the plant deviated significantly from the nominal model, thereby preventing the DOB from achieving the intended disturbance rejection performance, as shown in Figure 7. This large model uncertainty persists after t=60 s, rendering the conventional DOB ineffective in practice. In contrast, for the ADOB, the stability condition is satisfied over the entire operation period, as shown in the right column of Figure 8. The continuous update of the nominal model via parameter adaptation effectively reduces model uncertainty, thus maintaining the validity of the Q-filter design. Consequently, the ADOB achieves superior control performance compared with the conventional DOB using a fixed nominal model, which is consistent with the tracking error comparison presented in Figure 7.

## 5. Experimental Results with Three-DOF Robot Arm Manipulator

In the experimental results with the one-DOF electromagnetic actuator system, the multi-DOF control system with the ADOB demonstrated superior control performance even under artificially imposed model uncertainty. In this section, the same design procedure for the ADOB is applied to a higher-order dynamic system, which naturally involves parameter variation due to coupling dynamics among body segments.

To this end, localized controllers based on the DOB/ADOB structures were designed for each of the three joints, distal, middle, and proximal, relative to the base of the manipulator. Satisfactory tracking performance was first achieved via the DOB structure, and the performance was compared against the ADOB implementation, which demonstrated improved tracking accuracy, particularly at the proximal joint.

### 5.1. Experimental Setup

**Figure 9:**
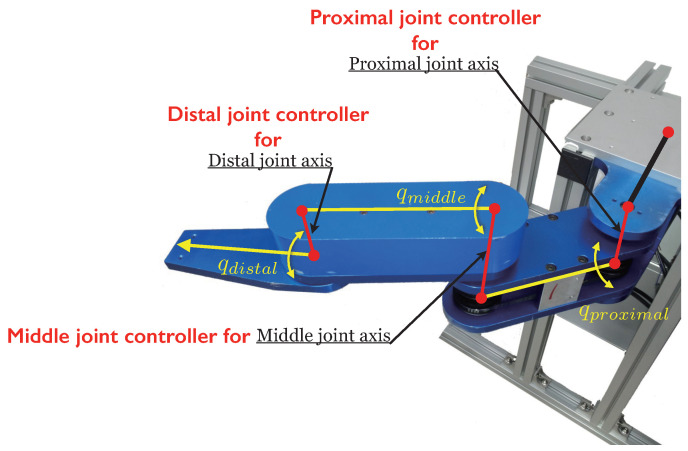
Experimental testbed 2; three-link robot arm manipulator controlled by localized controllers for each joint.

The proposed method was validated through experiments using a three-link robotic manipulator as depicted in Figure 9. Each link of the manipulator is driven by a 400 W AC motor from LG-OTIS Co. (Seoul, Republic of Korea), where motor angles are measured by encoders with a resolution of 8000 pulses per revolution. Joints and motors are coupled via belt drives, which introduce nonlinearities such as friction and backlash, while motors exhibit inherent nonlinearities in current-to-torque relationships. These nonlinear effects are intended to be rejected by the DOB throughout the experimental procedure.

Moreover, a multi-link manipulator can be characterized as a time-varying system because the inertia matrix D(q), Coriolis terms $C(q,\dot{q})$, and gravity effects G(q) depend on joint positions $q\in R^{3}$ and velocities $\dot{q}\in R^{3}$, where *q* and $\dot{q}$ denote generalized coordinates and velocities in the manipulator dynamics:

(29) $$D\left(q\right)\ddot{q}+C\left(q,\dot{q}\right)\dot{q}+G\left(q\right)=B\left(q\right)u+J{\left(q\right)}^{T}F_{ext}$$

Within this framework, J(q) denotes the Jacobian matrix and $F_{ext}$ denotes the external force. Consequently, changes in the state of each joint influence parameters of the nominal model, provided that an effective PAA updates the model in conjunction with motion of the robotic arm. In particular, when joints are managed by localized controllers, proximal joints experience more significant kinematic effects than distal joints. Furthermore, simultaneous motion across three joints induces more drastic parameter fluctuations due to dynamic coupling, since D(q), $C(q,\dot{q})$, and G(q) are generally non-diagonal matrices. In the experimental phase, each joint is controlled independently to evaluate tracking performance of the ADOB under varying kinematics, which effectively treats the Multi-Input Multi-Output (MIMO) system as a collection of SISO systems; thus, model uncertainty is introduced through the decoupling process. In addition, an AC motor can be regarded as a time-varying system over the full lifecycle due to mechanical wear, environmental factors such as ambient temperature, and demagnetization of internal magnets. Accordingly, parameter variation becomes unavoidable during long-term operation.

**Figure 10:**
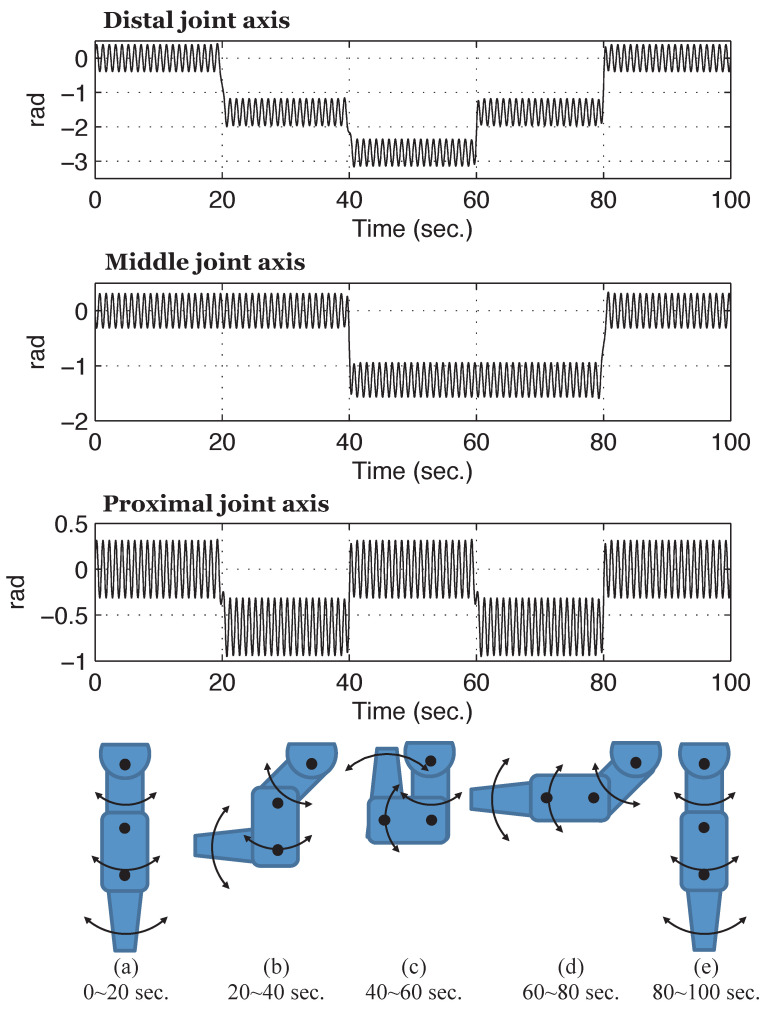
Reference trajectories for three joints of the robot arm.

In this experiment, a system with more rapid time-varying properties due to kinematic configuration was assumed; Figure 10 illustrates the reference trajectory of the joint angles. When all three joints were operated to follow the reference trajectories, the behavior of the three-link robotic arm evolved accordingly. The initial angle of each joint was defined as the forward outstretched posture illustrated in Figure 10a.

### 5.2. Controller Design

The control system was configured with a sampling frequency of 1 kHz. Based on a feasible linear structure of each joint, the initial nominal model was identified as follows:

(30) $$G_{n}\left(z\right)=\frac{1}{2875z^{2}-5276z+2041}$$

The same initial parameters were applied across all three joints. This initial nominal model was obtained through a fundamental frequency response analysis using the sine-sweeping method.

To evaluate the performance of the ADOB, measured tracking trajectories were compared against those of a conventional DOB that uses a fixed nominal model. First, a DOB-based controller with a fixed nominal model was implemented for reference tracking. Next, after integration of the PAA into the DOB as proposed, tracking performance of the resulting ADOB was assessed through comparative analysis against baseline performance of the DOB.

**Figure 11:**
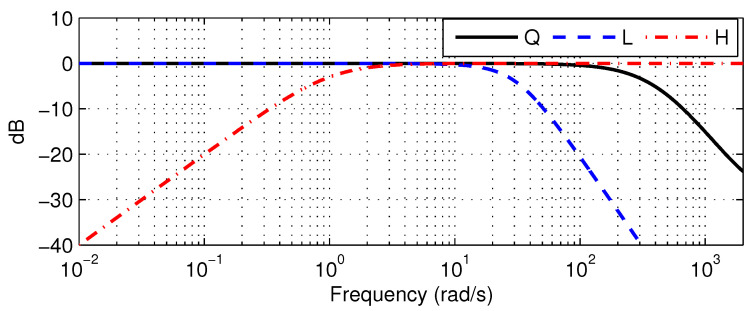
Frequency responses of the Q(z), L(z), and H(z).

Recall that Q(z) should be designed before implementation of the DOB architecture. Figure 11 illustrates the frequency response of the selected Q(z), defined as:

(31) $$Q\left(z\right)=\frac{0.1266}{z^{2}-1.288z+0.4149}$$

This transfer function represents an LPF with a cutoff frequency of 70 Hz. Bandwidth of Q(z) exceeds frequency components of the reference trajectory, which implies that the DOB remains effective across the spectral range of the designed trajectories. In addition, the DOB configuration with (31) was verified to satisfy the robust-stability condition via preliminary testing of the robotic arm at a specific posture, which corresponds to the initial nominal model in (30).

Subsequently, gains of the PD controller were selected based on the initial nominal model while considering gain and phase margins. The backward difference approximation was employed to derive the discrete-time PD controller as:

(32) $$C\left(z\right)=\frac{2025z-2000}{z}$$

The feedforward controller is automatically determined according to (23). Consequently, the multi-DOF control system integrated with the proposed ADOB is completed. While tracking control with the DOB demonstrated satisfactory tracking errors across all joints, performance of control at the proximal joint was inferior to that at the distal and middle joints due to model mismatch induced by the fixed nominal model in (30). This discrepancy was largely induced by shifts in kinematic configuration, particularly near the 40 s mark where the largest tracking error occurred during rapid configuration changes.

### 5.3. PAA in the ADOB

To design the ADOB, the PAA integrated with L(z) and H(z) is added to the existing DOB structure. Figure 11 illustrates the frequency responses of Q(z), L(z), and H(z). The filter L(z) mitigates rapid variation in parameters of the nominal model within the PAA while allowing slower transitions to pass. The LPF L(z) was established as:

(33) $$L\left(z\right)=\frac{4.696\times 10^{-4}z+4.696\times 10^{-4}}{z^{2}-1.95z+0.9512}$$

which yields a cutoff frequency of 4.94 Hz. Since links and motors are coupled via belt drives, significant nonlinearities arise due to friction and backlash. Consequently, since the disturbance information in the current setup predominantly consists of low-frequency components, H(z) is configured as an HPF:

(34) $$H\left(z\right)=\frac{0.999z-0.999}{z-0.999}$$

**Figure 12:**
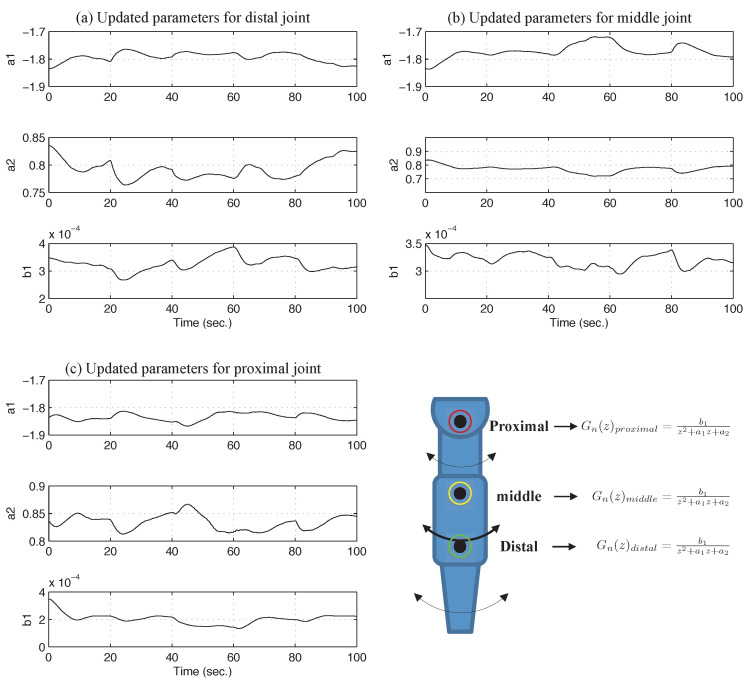
Updated model parameters of a three-joint robotic arm.

Figure 12 illustrates the experimental results of the time-varying model parameters estimated by the RLS algorithm based on the nominal model structure.

As illustrated in the figure, model parameters varied continuously during operation within the frequency range permitted by the designed L(z). This range influences the update rate of nominal parameters in the PAA and results in a gradual adaptation process. When the kinematic configuration of the three-link robotic arm underwent significant changes at 20, 40, 60, and 80 s, the model parameters updated by the PAA exhibited distinct convergence trends.

### 5.4. Quantitative Validation of Tracking Performance

The tracking performance of the proposed ADOB architecture was rigorously validated through a quantitative comparison. For this purpose, the $\ell_{2}$ norms of $e_{DOB}$ and $e_{ADOB}$ were calculated and summarized in Table 1 as a performance metric. It is observed that $∥e_{ADOB}{∥}_{2}$ for each joint of the robotic manipulator is consistently smaller than the corresponding $∥e_{DOB}{∥}_{2}$. In particular, the proximal joint exhibits a significant numerical discrepancy, which underscores the effectiveness of the adaptive approach in mitigating configuration-dependent uncertainty.

As the kinematic configuration of the three-link robotic arm varies, the inertia matrix relative to the proximal joint exhibits greater sensitivity than that of the other two joints under independent SISO control. This sensitivity leads to substantial variation in model parameters of the proximal joint and increases the model uncertainty. Persistence of this model mismatch renders the conventional DOB suboptimal in practice. In contrast, the ADOB attenuates model uncertainty by updating the nominal model based on parameters estimated via the RLS algorithm, thereby reducing the tracking error.

**Figure 13:**
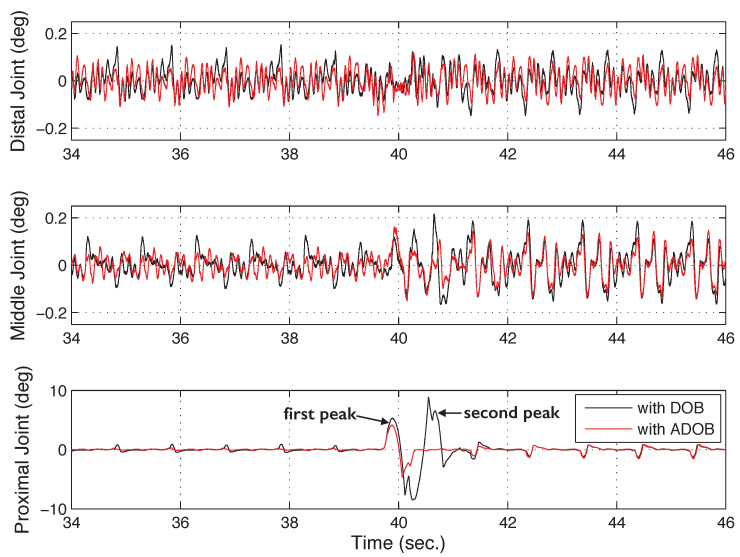
Comparison of tracking errors between the DOB and the proposed ADOB, highlighting the numerical reduction in residual errors.

Figure 13 illustrates the tracking errors for the DOB and the ADOB, showing a clear numerical gap in performance. Consistent with the reduction in the $\ell_{2}$ norm error, the ADOB maintains tighter error bounds than the conventional DOB. In particular, the maximum error at the proximal joint near 40 s in the DOB case is significantly mitigated when the ADOB is employed. While an initial peak remains similar during the early transient, a subsequent peak is effectively eliminated by the ADOB through real-time update of the nominal model.

Therefore, the ADOB reduces the tracking error induced by model mismatch that occurs when the DOB uses the fixed nominal model in (30). Furthermore, despite identifying a total of nine parameters across three joints in this experiment, the decentralized architecture distributed the computational load into independent low-order operations. Consequently, the total CPU occupancy of the ADOB logic remained consistently below 12% of the 1 ms sampling period, demonstrating the practical feasibility and scalability of the proposed framework for multi-DOF robotic systems.

## 6. Conclusions

This paper proposed the ADOB as a practical control methodology that integrates a DOB with the RLS-based PAA. Through functional division of labor and a virtuous cycle between the DOB and the PAA, the ADOB effectively addresses external disturbances, model uncertainties, and time-varying dynamics that inevitably arise in the control of physical systems. Nonlinearities and external interferences causing discrepancies between the actual plant behavior and the nominal model are estimated and immediately compensated for by the DOB in the form of disturbances. Simultaneously, the PAA continuously tracks long-term dynamic changes based on input–output data from the control loop, providing real-time tracking of structural model mismatches accumulated due to mechanical wear or changes in operating conditions. By updating the nominal model of the DOB with these identified parameters in real time, a robust functional positive feedback cycle between the DOB and the PAA is completed. However, since the DOB targets to “suppress” system variations while the PAA aims to “adapt” to them, functional interference inevitably occurs between the two algorithms as they possess opposing objectives regarding the same error phenomena. To address this issue, the proposed ADOB introduces a dual-filtering mechanism. In the frequency domain, a washout filter H(z) clearly separates the roles of the DOB and the PAA, while in the time domain, a smoothing filter L(z) is organically designed to mitigate the transient effects of abrupt parameter variations on the control loop.

The effectiveness of the proposed ADOB was validated through experiments on a one-DOF electromagnetic actuator and a three-DOF robot arm manipulator, where acquiring an accurate nominal model is challenging. Experimental results confirmed that the ADOB operates by distributing the functions of “suppression,” “adaptation,” and “separation and mitigation” to the DOB, PAA, and dual-filtering, respectively, in response to uncertainties arising from gear backlash, belts, and joint connections, as well as parameter variations due to time-varying dynamics. Consequently, it was verified that these three functions are organically integrated into a virtuous cycle. Furthermore, the proposed method demonstrated that a complex MIMO system, such as a robotic manipulator, can be controlled by independently decoupling it into individual SISO joint units. In particular, it was possible to effectively and individually address uncertainty factors of the links, including coupling uncertainties arising from interactions between joints, at each joint level. Although the proposed ADOB effectively responds to disturbances, model uncertainties, and time-varying dynamics, additional tuning may be required when system parameters fluctuate abruptly. For instance, a sudden change in the payload mass of a robotic manipulator can cause instantaneous shifts in equivalent parameters—such as effective inertia and gravity components—leading to severe model mismatches. Such rapid system changes violate the “slowly varying parameters” assumption, which is the mathematical foundation of adaptive control, and may consequently induce transient oscillations during the parameter identification process. Therefore, to prevent these rapid variations from being directly reflected in the adaptation process and to suppress excessive adaptive responses, it is necessary to organically tune the dynamic characteristics of Q(z), H(z), and L(z) so that the PAA can converge stably within the robust stability margin accommodated by the Q-filter of the DOB. Nevertheless, field practitioners can flexibly balance performance and robustness according to operating conditions through filter tuning alone, without the complex process of re-identifying the entire model. This realizes highly reliable model-based control that can be immediately implemented in industrial fields with minimal theoretical modeling, ultimately enabling a practical and field-oriented implementation of robust model-based control.

## Figures and Tables

**Table 1:** The $\ell_{2}$ norm of tracking error of the DOB and the ADOB.

Joint Number	${∥e_{\mathrm{DOB}}∥}_{2}$	${∥e_{\mathrm{ADOB}}∥}_{2}$
Distal joint	0.2807 rad.	0.2783 rad.
Middle joint	0.3038 rad.	0.2420 rad.
Proximal joint	3.4851 rad.	1.8310 rad.

## Data Availability

The raw data supporting the conclusions of this article will be made available by the authors on request.

## Abbreviations

The following abbreviations are used in this manuscript:

ADOB	Adaptive Disturbance Observer
DOF	Degree-of-Freedom
DOB	Disturbance Observer
EFS	Equivalent Feedback System
HPF	High-Pass Filter
LQR	Linear Quadratic Regulator
LTV	Linear Time-Varying
LPF	Low-Pass Filter
MPC	Model Predictive Control
MIMO	Multi-Input Multi-Output
PAA	Parameter Adaptation Algorithm
PT	Perfect Tracking
PE	Persistence-of-Excitation
PID	Proportional–Integral–Derivative
RLS	Recursive Least Squares
SISO	Single-Input Single-Output
SPR	Strictly Positive Real
ZOH	Zero-Order Hold
ZPETC	Zero Phase Error Tracking Control
